# Acknowledgement to Reviewers of *Pharmaceutics* in 2019

**DOI:** 10.3390/pharmaceutics12020088

**Published:** 2020-01-21

**Authors:** Pharmaceutics Editorial Office

**Affiliations:** MDPI, St. Alban-Anlage 66, 4052 Basel, Switzerland

The editorial team greatly appreciates the reviewers who have dedicated their considerable time and expertise to the journal’s rigorous editorial process over the past 12 months, regardless of whether the papers are finally published or not. In 2019, a total of 694 papers were published in the journal, with a median time to first decision of 16 days and a median time from submission to publication of 40 days. The editors would like to express their sincere gratitude to the following reviewers for their generous contribution in 2019:
Abatzoglou, NicolasLu, MingAbbina, SrinivasLu, ShenzhouAbbruscato, ThomasLu, WeiyueAbdelraouf, KamiliaLu, YanAbdel-Tawab, MonaLu, Zheng-RongAbuhelwa, Ahmad Y.Lucaciu, Constantin MihaiAcharya, GhanashyamLucas, Andrew T.Acree Jr., William E.Luciani, PaolaAderibigbe, Blessing Atim A.Lúcio, MarleneAdrover, AlessandraLuebbert, ChristianAghazadeh, AliLumay, GeoffroyAgrahari, VivekLunter, DominiqueAguilar-De-Leyva, AngelaLuo, RongcongAhmad, ZeeshanLux, JacquesAhmadzadehfar, HojjatLuzardo-Álvarez, AsteriaAhn, JaegyoonLyons, JohnAhn, Mi-JeongMa, XiangAimanianda, Vishu KumarMa, YuemingAizawa, TakafumiMa, Yunn-HwaAkagi, SatoshiMa, ZhiguoAkhlaghi, Seyedeh ParinazMaachani, Uday B.Alaimo, AlessandroMaafi, MounirAlaimo, SalvatoreMachelart, ArnaudAlanis-Lobato, GregorioMacphee, Iain AmAlba, MariaMadheswaran, ThiagarajanAlexander-Bryant, AngelaMaeda, TomokiAlfirevic, AnaMaeda, YukihideAl-Horani, Rami A.Maeng, Han-JooAliev, GjumrakchMaestrelli, FrancescaAl-Khattawi, AliMagda, JulesAlkilani, Ahlam ZaidMaggioni, DanielaAllegaert, KarelMaggo, SimranAllegra, SarahMagri, GiuliaAlmeida, AdelaideMahlin, DennyAlmeida, AntonioMajkowska-Pilip, AgnieszkaAlmoazen, HassanMajor, IanAlmoazen, HassenMajumder, PoulamiAl-Qahtani, MohammedMakky, AliAl-Sallami, HeshamMakvandi, PooyanAl-Tabakha, Moawia M.Malgieri, GaetanoAltieri, FabioMalic, SladjanaAmbrus, RitaMalik, DanishAnadón, ArturoMalik, Muhammad ImranAndrews, Allison M.Mallavia, RicardoAngela Barba, AnnaMalo De Molina, PaulaAngelico, RuggeroMamidi, NarsimhaAnisoara Peptu, CatalinaManak, Mark M.Anne-Claire, GrooManca, Maria LetiziaAnraku, MakotoManconi, MariaAntal, IstvánMandava, NandaAntimisiaris, Sophia G.Mandracchia, DeliaAntoniac, IulianManfredini, StefanoAntony, JaneMani, Ganesh KumarAparicio-Blanco, JuanManiruzzaman, MohammedApartsin, EvgenyMannell, HannaAramendia, IñigoMarć, Małgorzata AnnaArana, LideMarciello, MarziaAraújo, João M. M.Mares, MihaiArciello, AngelaMareș, MihaiArdejani, MaziarMareschi, KatiaArmanini, DecioMaria, Teresa M.R.Arote, Rohidas B.Marin, Jose JJArpagaus, CordinMarin, LuminitaArteaga, Jesús F.Márki, ÁrpádAscrizzi, RobertaMarkowicz-Piasecka, MagdalenaAsghar, SajidMaroni, PaolaAshaie, MaeirahMarson, DomenicoAshley, JonMartinez Rodriguez, FlemingAskes, SvenMartínez-García, MarcosAttia, F. MohamedMartinez-Lostao, LuisAyers, PhilMartino, SabataBabič, MichalMartins, José A.Babii, OlegMartins, NatáliaBabu, AnishMarto, JoanaBaby, AndreMaruthamuthu, MuraliBadea, NicoletaMasalova, Olga V.Baek, Jong-SuepMashima, RyuichiBai, Jane P FMaspoch, SantiagoBai, ShuhuaMassari, SerenaBaki, GabriellaMassella, DanieleBakowsky, UdoMasuda, SatohiroBalalaeva, Irina V.Masuo, YusukeBalas, MihaelaMatesic, LidiaBaldino, LuciaMatikonda, SiddharthBan, BhupalMatricardi, PietroBanciu, ManuelaMatta, Murali KrishnaBandarenka, Hanna V.Matters, Gail L.Bandari, SureshMaver, TinaBandyopadhyay, SulalitMazel, VincentBanga, AjayMazzoni, ChiaraBansal, ArvindMcardle, PatrickBansal, Kuldeep K.Mccallin, ShawnaBanti, Christina N.Mcconville, ChristopherBaptista, RafaelMccormick, Laura J.Barakh Ali, Sogra FathimaMcdaniel, JonathanBarbalexis, PanagiotisMcdonald, PeterBarbosa, Carlos MauricioMcdowell, ArleneBarbosa, Raquel De MeloMchale, AnthonyBarenholz, Yechezkel ChezyMchugh, KevinBarradas, Thaís NogueiraMcmurry, JonathanBarresi, AntonelloMees, MaartenBasit, AbdulMemon, TosifaBates, John T.Mendyk, AleksanderBattaglia, LuigiMeneguzzo, FrancescoBatzias, GeorgiosMenon, JyothiBazylińska, UrszulaMező, GáborBechelany, MikhaelMielańczyk, AnnaBellan, Leon M.Mihály, AndrásBelletti, BarbaraMilano, FrancescoBellich, BarbaraMilcovich, GesmiBeloor, JagadishMilczarek, MałgorzataBengani, LokendaMills, DavidBenson, HeatherMircioiu, ConstantinBeretta, Giovanni L.Mishra, Pawan KumarBergonzi, Maria CamillaMitchell, AndrewBernards, NicholasMitchell, MiguelBerthelsen, RagnaMitkin, Nikita A.Bettache, NadirMittal, Nivesh K.Bettencourt, Ana FranciscaMiyake, MasateruBeyer, WMiyamoto, HirotakaBhang, Suk HoMizrahi, BoazBianco, I.D.Mobed-Miremadi, MaryamBiedermann, ThomasMochel, Jonathan Paul MBiernasiuk, AnnaMochizuki, ShinichiBijak, MichalModi, NisargBiocca, SilviaMohammad, Abdul KhaderBiondi, MarcoMohammed, YousufBishop, RogerMohammed-Saeid, WaleedBlaabjerg, LasseMølhøj, MichaelBlanco-Fernandez, BarbaraMolinaro, RobertoBlencowe, AntonMoll, Gert N.Bleul, ReginaMondal, GoutamBobbala, SharanMonroe, Mary Beth BrowningBochicchio, SabrinaMontanari, ElitaBoddu, Sai H. S.Montenegro, LuciaBoekema, BoukeMonti, DanielaBogen, Kenneth T.Monti, Maria ChiaraBogni, AlessiaMoon, Geon DaeBoix-Montañés, AntonioMor, AmramBokias, GeorgiosMorales, JavierBoldyreva, ElenaMorandi, LucaBölükbas, DenizMorio, FlorentBonadies, IreneMoritz, MichałBonani, WalterMorris, John B.Bonferoni, Maria CristinaMourtas, SpyridonBono, NinaMoustafine, RouslanBoonen, MarielleMouzaki, AthanasiaBorrego-Sánchez, AnaMoyano-Mendez, Jose RamonBošković, PericaMu, QingxinBotrè, FrancescoMudalige, Thilak K.Bousbaa, HassanMukherjee, RajBraga, Susana SantosMukherjee, SudipBrambilla, DavideMuntimadugu, EameemaBrandl, MartinMura, SimonaBrayden, David J.Muralidharan, PriyaBrenza, TimothyMurnane, KevinBriedis, VitalisMuthu Karuppan, Mohan KumarBrockhoff, GeroMuthuramu, IlayarajaBrocks, DionMuthusamy, NagendranBrogi, SimoneMuttil, PavanBrooks, AmandaMyśliwiec, HannaBrozovic, AnamariaNa, Dong HeeBruni, GiovannaNag, Okhil KumarBrunnée, JuttaNagai, KatshitoBuchwald, PeterNagata, ToshiBudai-Szűcs, MáriaNaik, ShivangiBuddaseth, SalmaNair, Devatha P.Bulitta, JürgenNair, Praful R.Bunt, CraigNajlah, MohammadBurcynski, FrankNakanishi, TakeoBurga, RachelNakielski, PawelBurgalassi, SusiNam, Ki ChangBurley, Jonathan C.Nanaki, StavroulaBusardò, Francesco PaoloNanavati, CharviBusca, AureliaNand, AshveenBüsselberg, DietrichNatesan, ShanmugasundaramCacciatore, IvanaNawaz, MuhammadCaddeo, CarlaNaylor, AndrewCajal, YolandaNebbioso, MarcellaCalin, ManuelaNebot, Vicent JoséCalvignac, BriceNegri, DonatellaCameron, AngusNesamony, JerryCammas-Marion, SandrineNeubert, ReinhardCandiani, GabrieleNg, Wei LongCangkrama, MichaelNgamcherdtrakul, WorapolCannon, RichardNguewa, PaulCannon, Ronald E.Nguyen, Duc ThanhCapasso, RaffaeleNicholas T., BelloCardinale, JensNielsen, Line HagnerCarla, M. CaramellaNiemczyk, AgataCarmona, FranciscoNieves-Cintron, MadelineCarmona-Ribeiro, Ana M.Nikolakakis, IoannisCarpentier, RodolpheNitulescu, George MihaiCarter, KatharineNoh, KeumhanCasalini, TommasoNokhodchi, AliČasar, ZdenkoNonnemacher, Michael R.Casettari, LucaNoriyasu, KameiCassano, DomenicoNotario-Pérez, FernandoCastanheira, Elisabete M.S.Notni, JohannesCastiglione, ArcangeloNovell, AnthonyCastiglione, FilippoNsooudi, Nasim NsooudiCastillo, DanielNurieva, EvgeniyaCatanzano, OvidioO’Rear, EdgarCatucci, LuciaOba, MakotoCava, ClaudiaObata, YasukoCavalcanti Da Silva Filho, EdsonObermeier, BirgitCavalli, RobertaOcaña, JordiChae, HeeyoungOchowiak, MarekChalla, DilipOffer, StevenChan, Chun WaiOishi, AkioChan, K.L.Oka, SarangChandasana, HardikOkada, MasashiChang, Ming-WeiOkesola, BabatundeChang, Wei-JenOkour, MalekChang, Yan-ZhongOkuda, TomoyukiChar, Hyuk-JinOliveira, AndreiaChaudhari, SmrutiOliveira, J.VladimirChauhan, NeerajOmote, HiroshiCheaburu-Yilmaz, Catalina NataliaOng, Sang BingChen, DaiqinOnodera, RisakoChen, HaoqingOpaliński, ŁukaszChen, HongweiOtagiri, MasakiChen, JunOtero-Espinar, Francisco JavierChen, Kuo-YuOtsuka, MakotoChen, Yuan-ChuanOuyang, DefangCheng, Fong-YuPacelli, SettimioCheng, Nai-ChenPachauri, VivekChereddy, KiranPadrela, LuisChetoni, PatriziaPage, SusanneChetty, ManoranjenniPaik, Soon YoungChew, Sue AnnePalage, MarianaChiang, Po-ChangPalamà, IlariaChiang, Yi-TingPalermo, GiovannaChiesa, EnricaPaliwoda, DamianChina, ArnabPalugan, LucaChinnappan, MahendranPan, HaihuaChiriac, AuricaPan, XinChitescu, Carmen LidiaPang, JiayunCho, Cheong-WeonPapadimitriou, VassilikiCho, Hea-YoungPapadopoulou, LefkotheaCho, HyunahPapari, Gian PaoloCho, SungpilParayath, NehaChoi, Hyo-JickParis, JuanChoi, JonghoonParisi, LudovicaChoi, Seong-OPark, Chun-WoongChong, LiPark, Eun-JooChong, Parskson Lee GauPark, FrankChou, Shih-FengPark, Jeong-SookChow, Aviva Shing FungPark, Jin WooChow, Michael Y.T.Park, JuhyunChowdhury, Ezharul HoquePark, WooramChristensen, Jørn BParojčić, JelenaChristova, DarinkaPatching, SimonChronakis, Ioannis S.Patel, AshokChung, Seung WooPatel, HardikkumarCicero, ArrigoPathak, YashwantCirillo, GiuseppePathakoti, KavithaCiszewski, Wojciech MichałPathmasiri, WimalCoclite, Anna MariaPatil, Avinash J.Codacci-Pisanelli, GiovanniPatrinos, GeorgeCoelho, CarolinaPatterson, JenniferColeman, AnthonyPaudel, AmritColombo, GaiaPauer, WernerColuccia, MauroPaulmurugan, RamasamyConceição, JaimePaunesku, TatjanaConti, BicePearce, JoshuaConway, BarbaraPeeters, MarloesCordeiro, Ana SaraPeinado, María ÁngelesCórdoba, ManuelPein-Hackelbusch, MiriamCosco, DonatoPeltonen, LeenaCosta, PedroPenchev, HristoCouillaud, FranckPeng, ZhihongCourtenay, AaronPerche, FédéricoCoutinho, PaulaPerduca, MassimilianoCoutinho, Paulo José GomesPereira, Maria CarmoCova, TâniaPereira, PriscillaCraig, DuncanPereira-Leite, CatarinaCrona, DanielPerera, ManomiCrucho, CarinaPérez-Lozano, PilarCsoka, LeventePérez-Luna, Victor H.Cunha-Filho, MarcilioPerinelli, Diego RomanoCurtis, Anthony D.M.Perissutti, BeatriceCutrignelli, AnnalisaPersechino, SeverinoD’Alessandria, CalogeroPerteghella, SaraD’Amora, UgoPhan, Thi Tuong VyD’Apice, LucianaPiazzini, VieriDa Costa, Ana RosaPignatello, RosarioDahlgren, DavidPilli, NageswaraDalmoro, AnnalisaPiluso, SusannaDandawate, PrasadPincus, SethDangol, ManitaPintea, AdelaDanielak, DorotaPinteala, MarianaDanielsen, MichaelPinto Da Silva, LuísDas, Diganta B.Pinto Reis, CatarinaDash, RanjeetPinto, SandraDashevskiy, AndriyPippa, NatassaDate, AbhijitPirani, VittorioDatta, KausikPirianov, GrishaDavid, Sheba R.Pirzada, TahiraDavis, MarkPispas, StergiosDavis, TomPletzer, DanielDavoodi, PooyaPlotniece, AivaDe Aberasturi, Dorleta JiménezPokorny, MarekDe Bock, MarijkePollet, JeroenDe Caro, VivianaPolyakov, NikolayDe La Fuente, CesarPopat, AmiraliDe Luca, MichelePorebski, GrzegorzDe Melo-Diogo, DuartePoudel, BarunDe Miranda, Gustavo CabralPouliopoulos, AntoniosDe Oliveira, Paulo RenatoPrassl, RuthDe Paula, Haroldo César BeserraPravetoni, MarcoDe Paz, Antonio TaberneroProctor, RichardDe Paz, M. ViolanteProhens, RafelDe Robertis, MariangelaProkopowicz, MagdalenaDe Sousa, Célia TavaresProw, TralDe Stefano, LucaPrzybyłek, MaciejDe Villiers, Melgardt M.Puglia, CarmeloDe Zotti, MartaPushchina, EvgeniyaDea-Ayuela, María AuxiliadoraPutra, Okky DwichandraDegoul, FrançoiseQasim, MuhammadDel Pozo-Rodríguez, AnaQi, FengDel Re, MarziaQi, RongsuDelalande, AnthonyQi, ZhouDelgado, Ángel V.Quan, GuilanDelgado-Charro, M. BegoñaQuarta, AlessandraDelong, Robert K.Quiñones, Luis AbelDelouise, Lisa A.Quodbach, JulianDen Haan, Joke M.M.Rachid, OusamaDevoogdt, NickRadaram, BhaskerDhenge, RanjitRagno, GaetanoDi Carlo, Marta Di CarloRagusa, AndreaDi Maro, AntimoRai, PrakashDi Martino, PieraRaimi-Abraham, BahijjaDi Marzio, LuisaRais, RanaDi Paolo, AntonelloRajendran, Jagathesh ChandraDi Pasqua, Anthony J.Rajkowska, KatarzynaDiaferia, CarloRamasamy, MohankandhasamyDiaz-Rodriguez, PatriciaRamasamy, ThiruganeshDing, JianxunRamírez, DavidDinu-Pirvu, Cristina-ElenaRamos Cabrer, PedroDjuris, JelenaRamsköld, DanielDodou, KalliopiRancan, FiorenzaDoh, Kyung-OhRanganathan, ShivakumarDomb, AviRathinasabapathy, AnandharajanDong, XiaoweiRavaioli, StefanoDonnelly, RyanRavula, AbhigyanDorfman, RuslanReal-Oliveira, M. Elisabete C.D.Dory, DanielReffuveille, FanyDouroumis, DennisRegdon, GézaDrabczyk, AnnaRemedios Serrano, DoloresDrude, NataschaRibeiro, DanielaDu, JuanjuanRibeiro, HelenaDu, YongRibeiro, Viviana P.Duatti, AdrianoRice, Peter J.Dubbelboer, IlseRichardson, JosephDube, AdmireRichardson, NormanDudhipala, NarendarRigano, LuigiDugar, RohitRinaldi, FedericaDutta, KingshukRingshausen, Felix C.Düzgünes, NejatRistori, SandraDzmitruk, VolhaRiteau, NicolasEdagwa, Benson JRobert, JacquesEdginton, Andrea NRochfort, KeithEdina, PallagiRodallec, AnneEdiriweera, Meran KeshawaRodrigues, Célia F.Egan, DavidRodríguez Hermosa, Juan LuisEgan, PaulRodríguez-Yoldi, María JesúsEgito, Eryvaldo Sócrates TabosaRojas, SaraEgodawatte, ShaniRojo, LuisEiró, NoemíRoman, Andres DavidElaissari, AbdelhamidRong, MingqiangElhissi, AbdelbaryRonsard, LaranceEl-Masri, HishamRosado, CatarinaEloy, Josimar De OliveiraRosania, GusElshaer, AmrRosania, GustavoEmady, HeatherRose, KlausEmond, Claude.Rosière, RémiEndo, MakotoRossi, AlessandraEngelke, HannaRossi, FilippoEnosi Tuipulotu, DanielRossi, FilomenaEnrione, JavierRout, BhimsenEppard, ElisabethRozhkova, Elena A.Erdő, FranciskaRudd, SeanEritja, RamonRudra, ArnabEscargueil, AlexandreRyu, Ja-HyoungEspuelas, SocorroRyu, Jung SuEugster, PhilippeSaad, HosamEvans, CameronSabel, Bernhard A.Fabiano, AngelaSacher, StephanFagiolino, PietroSaenz Del Burgo, LauraFakhrullin, Rawil F.Saha, AchintoFalsini, SaraSalamanca, Constain H.Fan, WeizhengSaleem, ImranFang, JimSaleh, TawfikFang, LiangSaliev, TimurFang, MingxiSalis, AndreaFaria Ribeiro, Ana CristinaSalman, MootazFarrugia, BrookeSalmaso, StefanoFasinu, PiusSambuy, YulaFatouros, Dimitrios G.Samoylova, Tatiana I.Fay, FrancoisSánchez Crespo, MarianoFeczko, TivadarSánchez Navarro, AmparoFeczkó, TivadarSanchez, ElenaFelgueiras, HelenaSánchez-García, DavidFeng, Sheng-WeiSandri, GiuseppinaFenyvesi, FerencSanti, MelissaFerraz, RicardoSanti, PatriziaFerreira, Rui M.Santos De Almeida, TâniaFerrero, IvanaSapino, SimonaFigueiras, AnaSapudom, JiranuwatFigueiredo, Isabel VitóriaSara, ZalbaFilho, Marcílio CunhaSaraiva, M. Lúcia M.F.S.Fini, PaolaSaravanakumar, KandasamyFink, James B.Sarma, SauravFinlay, Warren H.Sarraguça, MafaldaFirlak, MelikeSathekge, MikeFischer-Fodor, EvaSato, HideyukiFlament, Marie-PierreSautto, Giuseppe A.Flores Johnson, Emmanuel AlejandroSaville, DorothyFocaroli, StefanoSayin, RidadeFogacci, FedericaScala, AngelaFologea, DanielScavello, FrancescoFonseca-Santos, BrunoScavo, Maria PrincipiaFonte, PedroSchaiquevich, PaulaFornaguera, CristinaSchindowski, KatharinaFortuna, AnaSchirhagl, RomanaFox, David T.Schneider, FelixFracasso, GiulioSchönthal, Axel H.Francés-Soriano, LauraSchoubben, AurélieFranco, Marina SantiagoSchumann, CananFrank, LuizaSchwartz, Stanley A.Freebairn, RossSedlaříková, JanaFrey, KathleenSegale, LorenaFriberg, LenaSeib, PhilippFroelich, AnnaSeidlitz, AnneFröhlich, EleonoreSeiner, Brienne N.Fromen, CathySenter, Peter DFuchs, HendrikSerwotka-Suszczak, AnnaFukuta, TatsuyaSgrignani, JacopoFumoto, ShintaroShah, Dhaval AFuruishi, TakayukiShah, RohanGadde, SureshShakya, AkhileshGagat, MaciejShankar, EswarGajdács, MárióShariare, MohammadGala, Rikhav PSharkawi, TahmerGalateanu, BiancaSharma, Amar DeepGalbis, ElsaShcharbin, DzmitryGanguly, SamitShelke, GaneshGao, ChunshengShen, GuofangGao, HuileSher, Yuh-PyngGarbayo, ElisaShi, LeiGarcia-Gallego, SandraShi, WenGarcia-Gonzales, CarlosShi, YangGarcia-Lopez, PatriciaShigdar, SarahGarcia-Pardo, JavierShimodaira, ShigetakaGarofalo, MariangelaShimomura, HirofumiGas, PiotrShimpi, ManishkumarGaspar, Maria ManuelaShin, Crystal S.Gately, Noel M.Shin, SoyoungGe, XiaodongShityakov, SergeyGellerman, GaryShojaei, ShahlaGeorgieva, RadostinaShokal, UpasanaGerogiorgis, DimitriosSideratou, ZiliGerratana, LorenzoSiegmund, WernerGeszke-Moritz, MałgorzataSignore, GiovanniGhadiri, MalihehSigurðsson, Hákon HrafnGhazanfari, LidaSilva, Margarida F. B.Ghica, Mihaela-VioletaSilva, Nuno H. C. S.Ghonaim, HassanSilva, SofiaGhori, Muhammad UsmanSilva, TaniaGhosh, SantoshSilvan, Miguel MansoGiannatsis, JohnSim, TaehoonGiansanti, LuisaSima, LiviaGikas, EvagelosSimone, ElenaGil, Jesus PerezSimos, YannisGillespie, James W.Singaraju, AdityaGiner-Casares, Juan J.Singh, ShaktiGiovagnoli, StefanoSinha, AbhijeetGiovannelli, PiaSinko, PatrickGlass, Beverley D.Sintim, HermanGoda, KatalinSiracusa, RosalbaGok, OzgulSiracusa, ValentinaGomez-Florit, ManuelSivasubramanian, KathyayiniGomez-Ramirez, RafaelSkalko-Basnet, NatasaGonçalves, Lídia M. D.Skiba, MohamedGonda, SandorSkirtach, AndreGong, HuaSkorik, Yury A.Gonzalez Alvarez, IsabelSkouras, AthanasiosGonzález-Rodríguez, María LSkwarczynski, MariuszGopalakrishnan, GopanSlütter, BramGorman, GregSobczak, MarcinGośliński, TomaszSobel, JackGoukassian, DavidSobotta, ŁukaszGoyanes, AlvaroSobue, TakanoriGracia, IgnacioSolanki, NayanGradoni, LuigiSolomon, MelaniGrandfils, ChristianSomani, SukrutGrassi, MarioSong, AnnaGrattoni, AlessandroSong, Im SookGray, Michael D.Sopper, SieghartGrice, Jeffrey E.Sorrenti, MilenaGrijalvo, SantiagoSoto, FernandoGrimaudo, Maria AuroraSovinco, FabioGrivel, Jean-ClaudeSpanakis, MariosGrodeland, GunnveigSprowl, Jason A.Grumezescu, Alexandru MihaiSrcic, StaneGruszecki, WieslawSreerama, SubramanyaGryczke, AndreasSrinivasan, Dinesh KumarGrzeskowiak, BartoszSrinivasan, SowmyaGuarino, VincenzoStahl, FrankGugliandolo, EnricoStaneva, DesislavaGuiot, CaterinaStanisz, BeataGuo, BaolinStavraka, CharaGuo, DandanStawny, MaciejGupta, BrijSteinbach-Rankins, JillGupta, PrachiStepensky, DavidHaddrell, Allen E.Stieger, BrunoHaemmerich, DieterStiriba, Salah-EddineHagenbuch, BrunoStoychev, GeorgiHama, SusumuSu, JingHamman, JoiasSu, YongchaoHan, FelicitySugano, KiyohikoHan, Hwa SeungSugiyama, HirokazuHan, SeunghoonSun, ChenHaneke, EckartSun, ConroyHaniu, HisaoSun, JingjingHao, JinsongSun, LemingHasan, AbsharSun, WeiHasanzadeh Kafshgari, MortezaSun, WujinHatziantoniou, SophiaSuresh, KuthuruHay, RodSutaria, DhruvitkumarHayashi, Jun-IchiroSutariya, VijaykumarHayashi, RyujiSuzuki, ToyofumiHe, HongliangSvensson, ElinHe, JingŚwiątek, ŁukaszHe, WeilueSwindle-Reilly, Katelyn E.Heath, TimothySzafraniec-Szczęsny, JoannaHeinämäki, Jyrki TapioSzekalska, MartaHens, BartSzilagyi, AndrasHerold, NikolasSzlęk, JakubHerraiz, Joaquín LópezSzweda, PiotrHertzler, RussellSzymańska, EmiliaHerwadkar, AnushreeTaboada Antelo, PabloHickerson, RobynTagad, HarichandraHinrichs, Wouter L. J.Tajber, LidiaHirayama, FumitoshiTakahashi, YukiHofmann, UteTakeda-Morishita, MarikoHoldsworth, CloviaTakeoka, ShinjiHomaeigohar, ShahinTallapaka, Shailendra B.Horhat, Florin GeorgeTampucci, SilviaHoskins, ClareTan, XuHsu, Fu-YinTang, YuanHu, JackTappa, KarthikHu, JinmingTaratula, OlehHu, LimingTashima, ToshihikoHu, Teh-MinTattikota, Sudhir GopalHua, XiaoqingTavakoli, JavadHuang, ChaoboTavangarian, FariborzHuang, Han HsiangTejada, Miguel ÁngelHuang, JiangengTekko, IsmaielHuang, WeiTelukutla, Srinivasa ReddyHuang, YingTeng, PengHuang, YuanyuThakur, Basant KumarHuang, YugangThakur, SachinHui, Patrick Chi-LeungThomas, Neil R.Huisinga, WilhelmThummuri, DineshHuntosova, VeronikaThurber, Greg M.Hwang, Sung-JooTian, JustinHyun, Dong ChoonTietze, RainerIaccino, EnricoTimmerman, PeterIatrou, HermisTing, Jeffrey M.Iglesias-Linares, AlejandroTirelli, NicolaIgnat, MariaTiwari, Rakesh K.Igne, BenoîtTomaiuolo, GiovannaIgnjatovic, NenadTomić, SimonidaIlyinskii, Petr O.Tomizaki, Kin-YaImperatore, RobertaTomuta, IoanInada, NataliaTong, ShengIncitti, TaniaTorigoe, KanjiroIoele, GiuseppinaTorrado, Juan J.Ionita, PetreTorrado-Santiago, SantiagoIrie, TetsumiTorre, Maria LuisaIshitsuka, YoichiTorres-Lagares, DanielIslam, NazrulTõugu, VelloIta, KevinTownley, Helen E.Ito, TaichiToyoda, YuIwasaki, TakashiTran, PhuongIzquierdo-Useros, NuriaTran, TtdJaiswal, Jagdish KumarTranquillo, ElisabettaJamrógiewicz, MarzenaTraverso, Carlo GiovanniJamwal, RohitashTripodo, GiuseppeJang, Mi-KyeongTrivedi, VivekJaramillo-Flores, MariaTsai, Ang-ChenJaszcz, KatarzynaTsai, Mei-LinJayant, Rahul DevTsai, Shiao-WenJedelská, JarmilaTsai, Wen-ChyanJee, Jun-PilTseng, Ching-LiJelonek, KatarzynaTseng, Kuang-WenJeon, JonghoTsiourvas, DimitrisJeong, Seong HoonTsitsilianis, ConstantinosJi, TianjiaoTsuji, Atsushi B.Ji, YuanhuiTsukamoto, TetsuoJia, DongyaTylkowski, BartoszJian, XingTzeng, Tzuen-RongJiang, ChengminUchida, SatoshiJiang, KeUekusa, HidehiroJiang, YanbingUeng, Yune-FangJiang, ZiwenUjhelyi, ZoltanJobin, Marie-LiseUndre, NasrullahJones, MarjorieUram, ŁukaszJović, DanicaUrbanek-Trzeciak, Martyna O.Juif, Pierre EricUrbanelli, LorenaJung, HyungilUrbinati, GiorgiaJung, JaehanUsui, KenjiKachrimanis, KyriakosVaidya, BhuvaneshwarKadam, AvinashVaidyanathan, BharatKalamvoki, MariaVaidyanathan, SriramKalhapure, Rahul S.Valenta, ClaudiaKalia, Yogeshvar N.Valente, JoanaKalogirou, OrestisVamanu, EmanuelKambayashi, AtsushiVan Bocxlaer, KatrienKambhampati, Siva PramodhVan Calenbergh, SergeKaminski, RafalVan Der Tol, ArjanKang, Han ChangVan Erp, PietKang, Myung JooVanan, Magimairajan IssaiKang, WonkuVanhaelen, QuentinKankala, Ranjith KumarVanic, ZeljkaKaralis, VangelisVanić, ZeljkaKarashima, MasatoshiVankayala, RavirajKarcz, DariuszVanscheeuwijck, PatrickKarolewicz, BożenaVarga, ZoltanKarpiński, Tomasz MVarum, FelipeKasperek, ReginaVasic, VesnaKastellorizios, MichailVassallo, AntonioKathuria, HimanshuVautier, SimonKatsila, TheodoraVávrová, KateřinaKatsumi, HidemasaVecchione, Dr RaffaeleKawai, MarikoVedagopuram, SreekanthKawakami, KohsakuVeeravalli, VijayabhaskarKazakov, SergeyVeiga, María-DoloresKeen, Justin MVenkataraman, ShrinivasKepinska, MartaVenkatesan, JayachandranKerai, LaxmiVibhute, SandipKesharwani, SiddharthVideira, Romeu A.Khadra, IbrahimVigani, BarbaraKhan, David R.Villarreal-Gómez, Luis JesúsKhan, Omar F.Viñas, MiguelKharissov, Boris IldusovichVllasaliu, DritonKharkar, PrathameshVoliani, ValerioKhoder, MouhamadVoronov, AndriyKhomane, Kailas S.Voshavar, ChandrashekharKhutoryanskiy, VitaliyVrečer, FrancKievit, ForrestWagemaker, Tais Aleriana LuconKillian, Manuela SonjaWagner, AlainKim, BohyungWalker, GregKim, BongjuWang, Jian-WenKim, Dong WukWang, JuanKim, Il WonWang, JunKim, Jae YoungWang, KaiKim, Keun-SikWang, QunKim, Min-SooWang, ShengKim, Pyung-HwanWang, Sheng-ShihKim, Seong-GonWang, Shi-BinKim, WankyuWang, TaiKim, Yu ChulWang, WeiKitchen, Scott G.Wang, WenxinKlajnert-Maculewicz, BarbaraWang, XiaofengKlar, AgnesWang, XiaohongKnapik-Kowalczuk, JustynaWang, XiaoweiKnez, ZeljkoWang, XiaoxingKnezevic, NikolaWang, XinKnopp, Matthias ManneWang, XinwenKo, Young TagWang, YuchenKobuchi, ShinjiWang, ZhijunKöhler, KarstenWani, Owies M.Kolanjiyil, Arun VargheseWarnken, ZacharyKolmar, HaraldWatts, AlanKolmas, JoannaWei, DongqingKolobov, Alexandr AlexandrovichWei, GangKoo, Hee-BeomWei, HuaKootala, SujitWeiss, Clemens K.Kopel, PavelWertz, PhilipKorhonen, OssiWhitehead, Debra E.Korostynski, MichalWibowo, DavidKorzhikova-Vlakh, EvgeniaWicha, SebastianKorzhikov-Vlakh, ViktorWilliams, GarethKostić, Aleksandar Ž.Williams, Noelle S.Kotla, NiranjanWilliamson, AdamKralj, SlavkoWin, Khin YinKrammer, Eva-MariaWinnicka, KatarzynaKrishnan, VinuWlaź, PiotrKrukiewicz, KatarzynaWoodward, Robert T.Kucuk, IsrafilWu, HuanKuehl, Philip J.Wu, Suh-ChinKujawska, MałgorzataWu, WeiKulbacka, JulitaWu, YunlongKumar Patel, SravanWu, Yu-TseKumar, MukeshWu, ZhangxiongKumar, NareshWuttke, StefanKumar, PradeepXia, TianKumar, PraveenXu, ChunKunda, NiteshXu, JianKuo, AndyXu, LuKurek, MateuszXu, RaymondKurrikoff, KaidoXu, TaoKusaczuk, MagdalenaXue, JiajiaKushwah, VarunYadavalli, TejabhiramKutwin, MartaYallapu, Murali MohanKwade, ArnoYang, FangKwiecień, IwonaYang, GuangKwon, Young M.Yang, Hung-WeiLabrou, NikolaosYang, Seung YunLai, FrancescoYang, YannanLai, Wing-FuYang, YongkangLakshminarayanan, RajamaniYang, Yueh-Hsun KevinLalatsa, AikateriniYang, ZheLallana, EnriqueYang, ZhenshengLameijer, Lucien N.Yao, JiayiLamprou, Dimitrios A.Yapp, Donald T.Lane, MajellaYazdan Panah, NimaLarhrib, HassanYazdi, ImanLarraneta, EnekoYe, ZhiweiLastovina, TatianaYen, Meng-ChiLaurenti, MarcoYeo, SynLaurini, ErikYeom, JunseokLayek, BuddhadevYi, TaoLeblond, JeanneYoo, Ji YoungLechanteur, AnnaYoon, In SooLee, AejuYounes, HusamLee, Bae HoonYu, Deng-GuangLee, Beom-JinYu, LuLee, Cheng-IYu, MeihuaLee, Chia-HungYu, WeilingLee, Dae HoYu, ZaiqunLee, Hye SukYuan, Wei-EnLee, JaehwiYuba, EijiLee, Jong BongYue, PengfeiLee, Joo HyoungYutani, ReikoLee, Mei-HwaZafar, MuhammadLee, SangkilZahínos, Emilio ViñuelasLee, Shiow-JuZaid, Abdel NaserLee, YongbokZaidi, SaheemLehocky, MarianZajdel, AlicjaLehr, Claus-MichaelZamostny, PetrLemercier, GillesZardán Gómez De La Torre, TeresaLeon, LorraineZarogoulidis, PaulLeporatti, StefanoZauscher, StefanLeung, Ada W.Y.Zdarta, AgataLeung, Shui YeeZelkó, RománaLi, FengZhang, Can YangLi, JiashenZhang, CanyangLi, JinboZhang, DianbaoLi, MengZhang, FanLi, MingqiangZhang, HaifeiLi, WenyiZhang, JiaxiangLi, XueZhang, JinjinLi, YingZhang, LiqiangLiagre, BertrandZhang, RujingLiang, XiaowenZhang, TaoLiao, Ai-HoZhang, WujieLiao, Shu-ChuanZhang, XiaohuiLiao, YonghongZhang, XuanLiaw, Chya-YanZhang, Yu (China)Libra, MassimoZhang, Yu (Cornell University, USA)Licciardi, MarianoZhang, Yu (Los Alamos National Lab, USA)Lim, Eun-KyungZhang, YueLin, BorenZhang, YumiaoLin, Hai-ShuZhang, ZiweiLin, XiaojieZhao, ChunxiaLin, ZhoumengZhao, QinjianLing, ChenZhao, YangLis Arias, Manuel J.Zhao, YongxinLiu, FeiZhao, ZongminLiu, HuolongZheng, XintingLiu, JinmingZheng, YangLiu, XifengZhou, JiangbingLiu, YuZhou, KunLiu, YuanZhou, YiqunLizal, FrantisekZhou, YubinLochhead, Jeffrey J.Zhu, CaihongLoganzo, FrankZhu, HongyanLombardi, TommasoZhu, JingenLombardi, Vincent C.Zhu, JunjieLombardo, DomenicoZhu, YishenLopalco, AntonioZiaee, AhmadLopes, Carla M.Ziemssen, FockeLópez-Cornejo, PilarZilberberg, NoamLorè, Nicola IvanZimoch-Korzycka, AnnaLoretz, BrigittaŻołek, TeresaLu, FangjiaZupancic, SpelaLu, Mei-ChinZydowsky, Thomas M

